# Lymphoid Leukemia in Bovines

**Published:** 1913-10

**Authors:** 


Lymphoid Leukemia In Bovines, *Ch. Aubèrtin and Th. Morel,* (*Arch. de Mal. du Coeur, des Vaisseaux, et du Sang,* 1913, VI, 201). According to the experience of the sanitary veterinarian of the Villette, only four or five cases of lymphadenia in calves and two or three in adult bovines were seen out of 450,000 animals slaughtered in 1911. The authors report two cases of characteristic lymphatic leukemia as proved by hematological and histological examination. They believe the cases of lymphadenia referred to by the veterinarians are of the same nature, or at least that lymphadenia may become converted into true lymphatic leukemia. One of the cases of lymphoid leukemia occurred in a five-year-old cow, the other in a two-months-old calf.

